# Emerging function and potential diagnostic value of circular RNAs in cancer

**DOI:** 10.1186/s12943-018-0877-y

**Published:** 2018-08-15

**Authors:** Xianglun Cui, Jianxun Wang, Zongjun Guo, Mengyang Li, Mingyu Li, Si Liu, Haoran Liu, Wenjing Li, Xunhua Yin, Jiaping Tao, Wenhua Xu

**Affiliations:** 1 Department of Inspection, The medical faculty of Qingdao University, Qingdao, 266003 China; 2 Center for Regenerative Medicine, Institute for Translational Medicine, Qingdao University, Qingdao, 266021 China; 3 Department of geriatric medical, Affiliated Hospital of Medical College, Qingdao University, Qingdao, 266003 China

**Keywords:** Circular RNA, Cancer, Biogenesis, Function, Biomarker

## Abstract

As a novel class of endogenous RNAs, circRNAs, have a covalently closed continuous loop, with neither a 5’to 3’polarity, nor a polyadenylated tail. Numerous circRNAs have been characterized by abundance, stabilization, conservation, and exhibit tissue/developmental stage-specific expression. Furthermore, circRNAs play vital roles in tumorigenesis and metastasis, such as functioning as a ceRNA or miRNA sponge, interacting with protein and encoding protein. Increasing evidence has revealed that it potentially serves as a required novel biomarker for cancer diagnosis. This review summarized the latest research on circRNAs, including its classification and biogenesis, mechanism and functions, as well as circRNAs in different cancers, as a potential biomarker.

## Background

As a novel class of long non-coding RNAs, circular RNAs (circRNAs) are widely expressed in the tree of life [1–3]. circRNAs have originally been considered as non-functional accidental by-products of aberrant splicing [4], which has not received enough attention. With the emergence of next-generation sequencing, especially RNA sequencing technology, numerous circRNAs have been found to be extensively expressed in eukaryotic cells. circRNAs are single-stranded transcripts derived from exons, introns, or intergenic regions that have a covalently closed continuous loop without a polyadenylated tail [5]. Due to the closed structure, circRNAs have been shown to be highly stable. Numerous circRNAs display evolutionary conservation, and the expression profiles are cell type- or developmental stage-specific.

Cancer is one of the most serious and life-threatening diseases, which has high morbidity and mortality worldwide, and a high frequency of metastasis and recurrence. Hence, there is an urgent need to identify potential biomarkers for prognosis predication, and determine new targets to design more powerful therapeutic approaches. Various studies have suggested that circRNAs are of great significance in tumorigenesis and metastasis, such as lung cancer [6, 7], colorectal cancer [8, 9], gastric cancer [10, 11], hepatocellular carcinoma (HCC) [12–15], breast cancer [16, 17], and so on. The present study summarized the latest research on circRNAs, including its classification and biogenesis, mechanism and functions, as well as circRNAs in different cancers, as a potential biomarker.

## Classification and biogenesis of circRNAs

According to composition, circRNAs can be classified into three categories: (a) ecircRNAs contain only exon sequences with 3′ → 5′-linked, which account for over 80% of discovered circRNAs [18–20]; (b) ciRNAs only consist of intron sequences with 2′ → 5′-linked intronic lariats, which are located in the nucleus [21, 22]; (c) EIciRNAs comprise of both exon and intron sequences with 3′ → 5′-linked, which are nuclear localized [23].

There is another principle of classification based on breakpoint location. According to location relationship of circRNAs with adjacent coding RNA, they are classified into five types: exonic, intronic, antisense, sense overlapping and intergenic. The first two kinds, like ecircRNAs and ciRNAs, composed of introns and exons. Antisense: derived from the opposite strand, whose sequences overlap with the linear mRNA. Sense overlapping: composed of same sequences as the linear mRNA, but not classified into exonic or intronic. Intergenic: consists of sequences located in noncoding region [24].

**Fig. 1:**
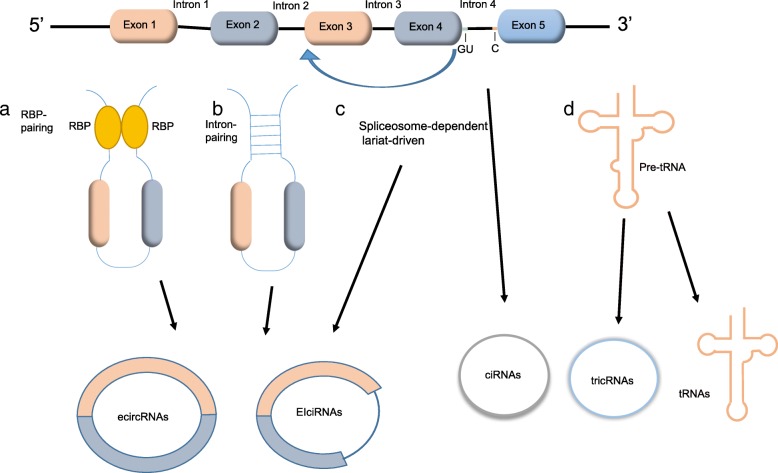
Biogenesis of circRNAs. **a** circRNAs formation can occur through RBPs mediated folding of the pre-miRNA. **b** the pairing between the two introns flanking the circularized exons, which have a complementary inverted sequence, can promote the backsplicing proceed in cis. **c** the back-splicing site promotes the joining of the downstream 5’donor sites with the upstream 3’acceptor sites. The lariat is subsequently processed by internal splicing, which finally results in the release of ecircRNAs or EIciRNAs. **d** tricRNA exon termini link with each other to form a mature tRNA, and intron termini are ligated together to form tricRNA

After being synthesized by RNA polymerase II, the precursor mRNAs (pre-mRNAs) are spliced and the introns are removed, alternatively joining the exons to generate linear mRNAs [25]. CircRNAs are also generated from pre-mRNAs through different mechanisms. There are three biogenesis mechanisms described below (Fig. 1).

### Spliceosome-dependent lariat-driven circularization

Exon circulation is spliceosome (or at least U1)-dependent as revealed by mutation of the 5′ splice sites [26]. In this model, the spliceosomes are assembled at back-splicing site to promote the joining of the downstream 5’donor sites with the upstream 3’acceptor sites. The lariat is subsequently processed by internal splicing, which finally results in the release of ecircRNAs or EIciRNAs. CircRNAs biogenesis and canonical splicing compete with each other [26]. Besides, the efficiency of backsplicing is much lower than that of linear splicing [27]. Backsplicing may occur post-transcriptionally, in which the circulation is an exon-containing lariat produced by exon skipping [18]. In addition, backsplicing can also occur co-transcriptionally, and the circular production from nascent mRNA does not need a polyadenylation signal [28]. The backsplicing involves single exon [18, 19] or several exons [20] with intervening introns.

Intron is nucleotide sequence between exons that is removed by RNA splicing during maturation of mRNA. However, some introns containing special motif escape from identification of debranching enzyme and form intron-derived ciRNAs. The essential motif consist of 7-nt GU-rich element located near the 5’splice site, and 11-nt C-rich element near the branch point [29]. As special ciRNAs, tricRNAs are derived from pre-tRNAs splicing and consist of intronic sequences. The biogenesis of tricRNAs conservatively exists in both archaea and eukaryotes relies on the splicing endonuclease complex, which can recognize the bulge-helix-bulge (BHB) sequence motif and cleave pre-tRNAs. Subsequently, exon termini link with each other to form a mature tRNA, and intron termini are ligated together to form tricRNA [30].

### Intron-pairing circulation

The pairing between two introns that flank the circularized exons, which have a complementary inverted sequence, can promote the backsplicing proceed in cis [31]. The paratactic intronic structure makes the splice donor close to the splice acceptor, and facilitates the nucleophilic attack and cleavage. One of the complementary repeats is Alu elements [32], which exist in more than 10% of the human genome. Alu elements derived from introns that flank circularized exons are more likely to complement, compared to other origins. Besides, complementary Alu elements are six-fold more likely present within flanking intron of circularized exons [1]. The competition between different-located reverse complementary sequences leads to production of diverse circRNA isoforms from a single gene. Furthermore, RNA pairing can occur at non-repetitive complementary sequences [31]. It has been reported that flanking sequence or structural complementarity is absent in *Drosophila* RNA circulation. Similarly, only a small proportion of circRNAs possess flanking intronic complementary sequences in rice. Moreover, complementary sequences not less than 30–40 nucleotides are able to assist circRNA biogenesis [33].

### RNA-binding proteins (RBP)-induced circulation

circRNA formation can occur through RBPs mediated folding of the pre-miRNA. RBPs, including Muscleblind (MBL) [26], Quaking (QKI) [34], Fused-in sarcoma (FUS) [35], are able to increase the rate of circulation by bridging relevant intronic sequences. The dimerization of RBPs, which binding with up- and downstream of the circularized exon, brings the 3′ and 5′ end of the circularized exons into close proximity and promotes their splicing. The flanking introns of circMBL contain conserved MBL binding sites. Moreover, the MBL interacts with its own pre-mRNA and stimulates cognate circRNA production. Conversely, mutation of the MBL binding sites evidently reduces circMBL production [26]. Regulated during epithelial-mesenchymal transition, QKI dynamically modulates the production of more than one-third of abundant circRNAs. Moreover, the RNA- and DNA-binding protein FUS binds to circularizing exon-intron junctions, and it regulates the production of 136 circRNAs in in vitro-derived mouse motor neurons. In the contrary, there are two RBPs: ADAR1 and DHX9. As a negative regulation factor, these reduce the formation of circRNAs. Furthermore, double-stranded RNA-specific adenosine deaminase (ADAR) has been found to diminish circRNA expression through the adenosine-to-inosine (A-to-I) editing activity, which makes RNA pairs anneal and reduces complementarity and backsplicing [32, 36]. Moreover, the nuclear RNA helicase DHX9 can interact with inverted-repeat Alu elements, downregulating Alu elements-induced intron pairing.

## Mechanism and function of circRNAs in cancer

**Fig. 2:**
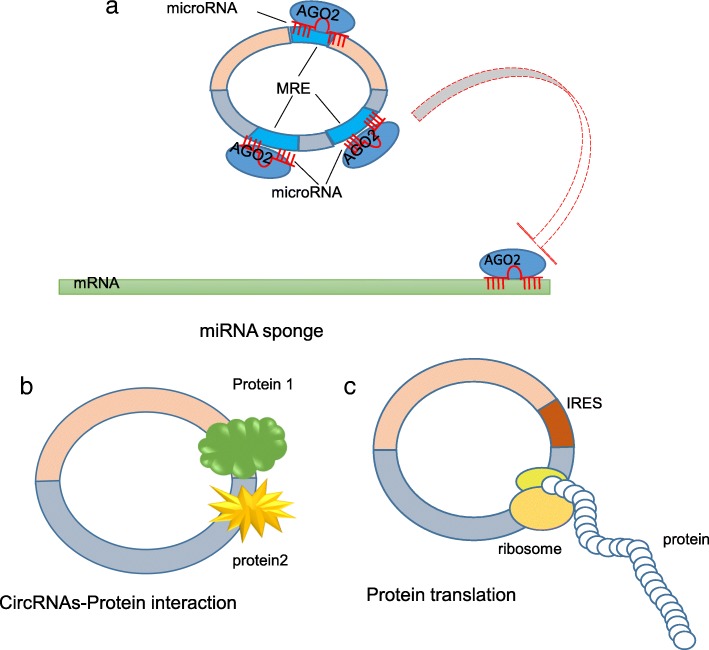
Functions of circRNAs. **a** miRNA sponge circRNAs can reduce miRNA activity by transcripts containing MREs, subsequently upregulating miRNA target expression. **b** CircRNAs-Protein interaction The interaction between circRNAs and proteins can facilitate the interaction of multiple proteins. **c** Protein translation circRNAs containing ORF and IRES have the potential to participant in translation

Recent studies indicate that circRNAs play a vital role in physiological and pathological processes at the post-transcription or transcription level. Here, we summarized the function and mechanism of circRNAs in cancer (Fig. 2).

### As competing endogenous RNAs or miRNA sponges

Competing endogenous RNA (ceRNA) is described as a complex post-transcriptional regulatory network mediated by sequestrating miRNAs [37, 38]. The hypothesis shows that miRNA activity can be reduced by transcripts containing miRNA response elements (MREs), subsequently upregulating miRNA target expression. Apart from mRNA, transcribed pseudogenes [38] and long noncoding RNA (lncRNA) [39], numerous studies have found that many circRNAs regulate miRNA network as ceRNAs [2, 40]. Furthermore, it has been shown that the majority of circRNAs are principally localized in the cytoplasm, suggesting that circRNAs may function as a miRNA sponge to sequestrate miRNAs (Fig. 2a). There are two characterized circRNAs,CiRS-7 and circSRY,verified this hypothesis. CiRS-7 (circRNA sponge for miR-7) contains more than 60 conserved miR-7 target sites, which are predominantly expressed in human and mouse brain [2],. CircSRY contains 16 putative miR-138 target sites that functions as miR-138 sponges [41].

Various circRNAs function as miRNAs sponges in tumorigenesis and progression. Hsa_circ_0012673 functions as a miRNA sponge of miR-22, which targets erb-b2 receptor tyrosine kinase 3 (ErbB3), promoting lung adenocarcinoma (LAC) cell proliferation [42]. As a tumor suppressor, circLARP4 is downregulated in gastric cancer (GC) tissues, suppressing gastric tumorigenesis and progression by sponging miR-424 and increasing LATS1 expression [10]. WJ Huang et al. demonstrated that hsa_circ_0000977 interacts with has-miR-874-3p, and subsequently promotes the expression of PLK1 in pancreatic ductal adenocarcinoma cancer [43]. In hepatocellular carcinoma, circHIPK3 functions as a miRNA sponge of miR-124, which sequentially upregulates the expression of AQP3, and promotes cell proliferation and migration [12]. Both circGFRA1 and GFRA1 are upregulated in triple negative breast cancer (TNBC), and circGFRA1 functions as a ceRNA to regulate GFRA1 expression by decoying miR-34a [16]. Chengdi Yang observed that circ-ITCH suppressed the aggressive biological behaviors of bladder cancer (BCa), and upregulated the expression of p21 and PTEN through decoys miR-17 and miR-224 [44]. The expression of circ-SHKBP1 is elevated in glioma-exposed endothelial cells (GECs), which functions as a ceRNA via the miR-544a/FOXP1 or miR-379/FOXP2 pathway [45]. Furthermore, L Chen et al. discovered that circRNA_100290 was elevated in OSCC tissues, which upregulates CDK6 expression through decoying miR-29b family members, playing a crucial role in OSCC progression such as tumor invasion and metastasis [46]. Upregulated circUBPA2 promotes osteosarcoma growth and inhibits apoptosis by downregulating the expression of miR-143, consequently raising the expression of anti-apoptosis Bcl-2 [47].

### Protein translation

Although defined as a subclass of non-coding RNAs, increasing evidence have demonstrated that circRNAs have potential to participate in translation [48, 49] (Fig. 2b). It can be presumed that a protein-translated circRNA when some of the following features are present: (A) circRNAs have ORF with sufficient length, which is essential for linear mRNA protein translation; (B) it has the spanning backsplicing junction ORF; (C) some of the necessary regulation elements are present to the translation initiation upstream ORF, such as the internal ribosome entry site (IRES) element, and the N6-methyladenosine (m6A) modifications near the start codon [50].

Recently, at least two cases offer important evidence for the existence of the translation of endogenous circRNA-encoded peptides: circFXBW7 and cricSHPRH. These are of great significance in tumorigenesis and progression. Y Yang et al. revealed that circFXBW7 can encode a novel 21-KDa protein, which was named, FBXW7-185aa. A dual luciferase vector system constructed with full length or truncated putative circ-FBXW7 IRES sequences was used to test for IRES activity. The result revealed that only the full length circFBXW7 IRES group can induce the highest Luc/Ruc activity. Next, the circ-FXBW7 vector and other control vectors were transfected into human cells, and FBXW-185aa was detected by a specific antibody and liquid chromatography tandem-mass spectrometry. The result suggests that circ-FBXW7 has the potential to encode a novel protein. The protein FBXW7-185aa functions as a tumor suppressor by competitively binding with USP28, and preventing USP28 binding to FBXW7α, subsequently inhibiting USP28-induced c-myc stabilization. Circ-FBXW7 and FBXW7-185aa were downregulated in glioblastoma, and suppress proliferation and cell cycle acceleration [51]. Another example is circSHPRH, which is generated by back-splicing exons 26–29 from the SHPRH gene, and encodes a 17 KDa protein SHPRH-146aa. SHPRH-146aa protects the full-length SHPRH from DTL-induced ubiquitination, while the latter sequentially ubiquitinates proliferating cell nuclear antigen (PCNA) as an E3 ligase. Both circSHPRH and SHPRH-146aa were downregulated in glioblastoma, which suppress cell proliferation and tumorigenesis [52]. In the summary, both circ-FBXW7 and circ-SHPRH contain the spanning junction ORF and IRES, and play an essential role in glioma tumorigenesis by encoding a protein.

### CircRNA-protein interaction

There are conserved, protein-binding sequences in some circRNAs which can be demonstrated by the co-localization of circRNAs and proteins. The interaction between circRNAs and proteins can regulate transcription of parental genes, facilitate the interaction of multiple proteins, as well as alter the subcellular localization of proteins. CircRNAs can function as a protein scaffolding to facilitate contact between two or more proteins (Fig. 2c). CircFoxo3 is perhaps the best example, which binds with cyclin-dependent kinase inhibitor 1 (p21) and cyclin-dependent kinase 2 (CDK2), forming a ternary complex. P21 can inhibit CDK2 activity and act as a regulator of cell cycle progression at the G1 and S phase. Circ-Foxo3 can facilitate the interaction between p21 and CDK2, resulting in the inhibition of the cell cycle [53]. Q Yang et al. observed that circAmotl1 and c-myc co-localizes to the nucleus, and the interaction between these two induces c-myc nuclear translocation and prevents degradation. CircAmotl1 is significantly increased in breast cancer and promotes tumorigenesis by interacting with c-myc [17].

### Other functional mechanism

Cancer-associated chromosomal translocations not only encode oncogenic fusion proteins, but also produce fusion circRNAs. J Guarnerio et al. found two aberrant f-circRNAs produced by chromosomal translocation: f-circM9 and f-circPR. These are correlated with the tumorigenesis and progression of hematological tumors. The expression of f-circRNAs in cancer cells is of great significance to cell survival and therapy resistance [54]. In addition, S Tan et al. discovered that endogenous F-circEA derived from EML4-ALK fusion gene was existed in H2228 cells with the EML4-ALK variant 3b translocation. Furthermore, F-circEA is not only existed in NSCLC tissues but also in the plasma of the patients with the EML-ALK translocation [7].

#### CircRNAs in cancer

Numerous circRNAs have been found to be dysregulated in tumor tissues, playing oncogenic or tumor-suppressor roles. Growing evidence show that circRNAs are significantly correlated with malignant behavior of tumor cells and clinical stage. Here, we summarized recent studies about regulatory networks and vital function of circRNAs in several mortal cancer (Table 1).

**Table 1:** summary of the expression and signaling pathways of circRNAs involved in cancer

Cancer type	CircRNA	Funtion	Expression in tumors	References
Lung adenocarcinoma	hsa_circ_0012673	promotes LAC cell proliferation and tumor growth by decoying miR-22	Up	[42]
	hsa_circ_0013958	promotes cell proliferation, migration and invasion by sponging miR-134	Up	[57]
	circPRKC1	positively correlated with tumor size and TNM stage, promotes cell proliferation and migration by regulating miR-545/589	Up	[6]
	circRNA_102231	associated with the advanced TNM stage, lymph node metastasis and poor overall survival, promotes cell proliferation and invasion ability	Up	[58]
	has_circ_0007385	promotes the proliferation activity, colony-forming ability, migration and invasion of NSCLC cells in vitro and the tumor growth in vivo by decoying miR-181	Up	[59]
	circ0006916	inhibits cell proliferation by regulating cell cycle progression	Down	[60]
	f-circEA	promotes cell migration and invasion	Up	[7]
	circFARSA	promotes cell migration and invasion by sponging miR-330-5p and miR-326	Up	[63]
	hsa_circRNA_103809	promotes cell proliferation and invasion in vitro and tumor growth in vivo by sponging miR-4302	Up	[61]
	circFADS2	correlated with advanced TNM stage, lymph node metastasis, poor differentiation and shorter overall survival of NSCLC patients. promotes cell proliferation and invasion ability	Up	[62]
Colorectal cancer	circHIPK3	promotes cell proliferation, and colony-forming ability, migration and invasion by decoying miR-7 invasion by decoying miR-7	Up	[8]
	circCCDC66	promotes cancer cell proliferation, migration and metastasis in vitro and in vivo by sponging miRNA-33b and miR-93	Up	[9]
	ciRS-7	positively correlated with advanced tumor stage, tumor depth and metastasis by downregulating miR-7	UP	[64]
	circBANP	related with cell proliferation and colony formation ability	Up	[65]
	hsa_circ_0014717	correlated with TNM stage, distal metastasis, and prognosis of CRC patients, antagonizes cell proliferation and colony-forming ability, as well as induces cell cycle arrest at G0/G1 phase	Down	[66]
Gastric cancer	has_cric_0000096	inhibits gastric cancer proliferation and migration by suppressing cyclin D1, CDK6, MMP-2 and MMP-9	Down	[68]
	circPVT1	promotes cell proliferation by sponging miR-125	Up	[11]
	circLARP4	Impairs GC cell proliferation and invasion, associated with tumor size, lymphatic metastasis and the therapeutic outcomes of GC patients by regulating miR-424	Down	[10]
Pancreatic cancer	hsa_circ_0000977	associated with poor prognosis in PDAC patients, promotes cell proliferation and colony formation ability by sponging hsa-miR-874-3p	Up	[43]
	circ-LDLRAD3	associated with venous invasion, lymphatic invasion and metastasis	Up	[70]
	circPDE8A	correlated with lymphatic invasion, T factor and TNM stage, promotes migration or invasion, cell proliferation and EMT	Up	[71]
Hepatocellular carcinoma	circMTO1	correlates with the poor survival of patients, inhibits cell proliferation and invasion by decoying miR-9	Down	[13]
	circARSP91	AR suppresses circARSP91expression by upregulating the expression of ADAR1	Down	[14]
	cSMARCA5	correlated with advanced tumor stage, tumor size and the presence of microvascular invasion through sponging miR-17-3p an miR-181b-5p	Down	[15]
	circ_0067934	promotes cell proliferation and metastasis in vitro and in vivo by regulating miR-1324	Up	[72]
	circRNA_100,338	correlated with decreased cumulative survival rate, increased vascular invasion and lung metastasis in HCC patients by suppressing miR-141-3p	Up	[73]
	circHIPK3	promotes the proliferation and migration of HCC cells, as well as xenograft tumor growth in vivo by downregulating miR-124	Up	[12]
	circC3P1	represses the migration and invasion of HCC cells in vitro, and inhibits HCC cells proliferation and lymphatic metastasis in vivo by sponging miR-4641	Down	[74]
Breast cancer	circGFRA1	correlated with tumor size, TNM staging, lymph node metastasis and histological grade, promotes cell proliferation and the colony-forming ability by decoying miR-34a	Up	[16]
	circAmotl1	increases invasive capacity, reduces number of apoptosis cells, and augmentes tumor-forming capacity by promoting c-myc nucleus-translocation	Up	[17]
	circRNA-000911	suppresses the invasive capacity and proliferation, reduces colony formation ability and elevates the proportion of apoptotic cells by sponging miR-449a	Down	[75]
	cricABCB10	promotes cell proliferation and colony formation capacity by downregulating miR-1271	Up	[76]
	hsa_circ_0011946	correlated with RFC3 expression, promotes migration and invasion of MCF-7 cell	Up	[77]
Bladder cancer	circ-ITCH	suppresses cell proliferation, migration and invasion in vitro, as well as tumorigenesis in vivo, through sponging miR-17 and miR-224	Down	[44]
	circRNA-MYLK	increases the tumorigenicity of BC cells, distinct metastatic lesions in mice lungs and tumor microvessels by decoying miR-29a/	Up	[79]
	circHIPK3	suppresses the aggressiveness and metastasis of bladder cancer cells in vitro and in vivo by targeting miR-558	Down	[80]
	Cdr1as	inhibits the migration and invasion capabilities, as well as induces cell cycle arrest	Down	[81]
Glioblastoma	circ-FBXW7	associated with glioblastoma patient overall survival, inhibits proliferation and cell cycle acceleration by encoding FBXW7-185aa	Down	[51]
	circSHPRH	encodes SHPRH-146aa which can protect SHPRH and reduce proliferation and the malignant phenotype	Down	[52]
	circ-SHKBP1	promotes the viability, migration and tube formation of glioma-exposed endothelial cells by sponging miR-544a or miR-379	Up	[45]
	cric-TTBK2	promotes cell proliferation, migration and invasion, but inhibits the apoptosis of glioma cells by regulating miR-217	Up	[82]
Other cancers				
Oral squamous cell carcinomas	circRNA_100290	promotes cell proliferation in vitro and the growth of tumors in vivo by decoying miR-29b	Up	[46]
	circDOCK1	inhibits cell apoptosis by sponging miR-196a-5p	Up	[83]
Osteosarcoma	cricUBAP2	correlated with tumor stages, promotes cell proliferation in vitro and cell growth in vivo by downregulating miR-143	Up	[47]
	circ-NT5C2	correlated with tumor volume and weight in a mice model, promotes cell proliferation and invasion in vitro by decoying miR-448	Up	[84]
	circRNA_0009910	promotes cell proliferation and inhibits apoptosis by sponging miR-449a/IL6R	Up	[85]
	has_circ_0001564	promotes cell viability and the colony formation vitality by decoying miR-29c-3p	Up	[87]
	circNASP	promotes the proliferation and invasion of OS cells by sponging miR-1253	Up	[86]
Hematological tumors	f-circM9, f-circPR	contributes to cellular transformation, and promotes cell viability and resistance upon therapy	Up	[54]

### Lung adenocarcinoma

Lung cancer is the leading cause for cancer-related death worldwide, and non-small cell lung cancer accounts for more than 80% of all lung cancer cases [55, 56]. LAC is the most common type in recent decades. The expression of has_circ_0012673 is significantly increased in LAC tissues, promoting LAC cell proliferation and tumor growth via the hsa_circ_0012673/miR-22/ErbB3 axis [42]. X Zhu et al. observed that cell proliferation was suppressed, cell apoptosis was induced, and cell migration and invasion was inhibited after silencing hsa_circ_0013958 [57]. In addition, Qiu et al. demonstrated that elevated circPRKCI promoted cell proliferation and migration through the circPRKCI-miR-545/589-E2F7 axis, positively correlated with tumor size and TNM stage. Treatment of si-circPRKCI significantly inhibited growth of SPC-A1-derived tumor xenografts and patient-derived tumor xenografts in vivo [6]. Similarly, circRNA_102231 promotes lung cancer cells proliferation and invasion ability in vitro. Moreover, increased circRNA_102231 is significantly associated with the advanced TNM stage, lymph node metastasis, and poor overall survival of lung cancer patients [58]. Hsa_circ_0007385 is up-regulated in NSCLC tissues and cells, acting as a sponge of miR-181. Hsa_circ_0007385 silencing suppresses the proliferation activity, colony-forming ability, migration and invasion of NSCLC cells in vitro and inhibits the tumor growth in vivo [59]. X Dai et al. discovered that circ0006916 was decreased in lung cancer cells and tissues, and inhibited cell proliferation by regulating cell cycle progression but not apoptosis [60]. F-circEA increases cell migratory and invasion ability, and dose not participate in cell proliferation and colony formation [7]. In addition, circFARSA, hsa_circ_103809, circFADS2 also play significant roles in lung cancer progression [61–63].

### Colorectal cancer

Colorectal cancer (CRC) is the third most frequent cause of cancer-related death in America [56]. CircHIPK3 knockdown significantly inhibits cell proliferation, and colony-forming ability, migration and invasion. Likewise, circHIPK3 silencing suppresses CRC growth and metastasis in xenograft animal models, and exhibits an additive effect on tumor repression [8]. CircCCDC66 serves as a miRNA sponge of miRNA-33b and miR-93, promoting cancer cell proliferation, migration and metastasis in vitro and in vivo [9]. In addition, ciRS-7 is overexpressed in CRC tissues, and positively correlated with advanced tumor stage, tumor depth and metastasis in CRC patients [64]. M Zhu et al. discovered that the proliferation and colony formation ability of the si-circ-BANP group was markedly repressed, when compared with negative control group [65]. Hsa_circ_0014717 is down-regulated in CRC tissues compared with adjacent normal tissues and closely correlated with TNM stage, distal metastasis, and prognosis of CRC patients. Moreover, hsa_circ_0014717 overexpression significantly antagonizes cell proliferation and colony-forming ability, as well as induces cell cycle arrest at G0/G1 phase [66].

### Gastric cancer

In 2012, gastric cancer (GC) ranks third in cancer-related deaths and fourth common gastrointestinal malignancies worldwide [67]. P Li et al. found that has_cric_0000096 was significantly reduced in gastric cancer tissues and cells. It can inhibit gastric cancer proliferation and migration by suppressing the expression levels of cyclin D1, cyclin-dependent kinase (CDK) 6, matrix metalloproteinase (MMP)-2 and MMP-9 [68]. The expression of circPVT1 is upregulated in GC tissues, when compared with matched normal tissues, and promotes cell proliferation, having the potential to serve as an independent prognostic indicator [11]. As a tumor suppressor, circLARP4 is downregulated in GC tissues, and impairs GC cell proliferation and invasion. In addition, the expression level of circLARP4 is associated with tumor size, lymphatic metastasis and the therapeutic outcomes of GC patients [10].

### Pancreatic cancer

Recently, the incidence and mortality of pancreatic cancer increase every year, worldwide. It is the 7th and 4th leading cause of mortality among all malignancies in China [69] and the United States [56]. WJ Huang et al. demonstrated that has_circ_0000977 is upregulated in pancreatic ductal adenocarcinoma (PDAC) tissues, and is associated with poor prognosis in PDAC patients. Hsa_circ_0000977 silencing suppresses cell proliferation, reduces colony formation ability, and induces G1/S arrest [43]. The expression of circ-LDLRAD3 is increased in both pancreatic cancer tissues and plasma in patients with pancreatic cancer. It is correlated with venous invasion in patients with pancreatic cancer [70]. Furthermore, circ-PDE8A is significantly correlated with lymphatic invasion, T factor and TNM stage. It promotes migration or invasion, cell proliferation and EMT via the miR-338/MACC1/MET pathway [71].

### Hepatocellular carcinoma

As the fifth leading cause of mortality from cancer-related diseases worldwide, hepatocellular carcinoma accounts for approximately 80% of primary liver cancers [56, 67]. CircMTO1 is downregulated in HCC tissues, and correlated with the poor survival of patients. After silencing circMTO1, the level of cell proliferation and invasion is significantly increased, and the percentage of apoptosis is reduced in vitro and in animal model prepared by transplanting human HCC tissues [13]. L Shi et al. revealed that androgen receptor (AR) could regulate circRNA expression in HCC by upregulating the expression of ADAR1, which enables the suppression of RNA circulation. The AR/ADAR1/circARSP91 axis is essential to HCC initiation and gender disparity. cSMARCA5 is decreased in HCC tissues, and correlated with aggressive biological behaviors, such as poorer tumor differentiation, advanced tumor stage, tumor size and the presence of microvascular invasion [15]. Circ_0067934 is highly expressed in HCC tissues, when compared with adjacent normal tissues, and promotes cell proliferation and metastasis in vitro and in vivo via the miR-1324/FZD5/Wnt/b-catenin axis [72]. G Chen et al.discovered that circHIPK3 promoted the proliferation and migration of HCC cells, as well as xenograft tumor growth, in vivo [12]. The ectopic expression of CircRNA_100,338 is correlated with decreased cumulative survival rate, increased vascular invasion and lung metastasis in HCC patients [73]. As a tumor suppressor, circC3P1 overexpression represses the migration and invasion of HCC cells in vitro, and inhibits HCC cells proliferation and lymphatic metastasis in vivo [74].

### Breast cancer

Breast cancer is one of the leading reasons of cancer-related mortality, and the most frequent cancer that occurs in women worldwide [56, 67]. The expression level of circGFRA1 is correlated with tumor size, TNM staging, lymph node metastasis and histological grade. The downregulation of circGFRA1 markedly impairs the proliferation potential and reduces the colony-forming ability of TNBC cells. Furthermore, the apoptosis of TNBC cells is promoted, and tumor growth is decreased in vivo upon circGFRA1 silencing [16]. Q Yang et al. demonstrated that cricAmotl1 promotes breast cancer progression, which is manifested by increased invasive capacity, reduced number of apoptosis cells, and augmented tumor-forming capacity [17]. H Wang et al. reported that circRNA-000911 suppressed the invasive capacity and proliferation of circRNA-000911-transfected cells. In addition, circRNA-000911 markedly reduces colony formation ability and elevates the proportion of apoptotic cells [75]. CricABCB10 is significantly upregulated in breast cancer tissues. In circ-ABCB10 knockdown cells, apoptosis is enhanced, and cell proliferation and colony formation capacity is suppressed [76]. Furthermore, has-circ-0011946 is significantly up-regulated in breast cancer and different breast cancer cell lines. The expression of has-circ-0011946 is positively correlated with RFC3 expression, silencing of has_circ_0011946 inhibits migration and invasion of MCF-7 cell [77].

### Bladder cancer (BCa)

BCa is the ninth most common carcinoma with high morbidity and mortality worldwide [78]. C Yang et al. found that circ-ITCH was reduced in BCa tissues and cell lines, suppressing cell proliferation, migration and invasion in vitro, as well as tumorigenesis in vivo, through the circ-ITCH/miR-17, miR-224/p21, and PTEN axis [44]. CircRNA-MYLK activates the VEGFA/VEGFR2 and Ras/ERK signaling pathways. As a potential oncogene, it increases the tumorigenicity of BCa cells, distinct metastatic lesions in mice lungs and tumor microvessels [79]. In addition, circHIPK3 regulates heparanase by targeting miR-558, sequentially suppressing the aggressiveness and metastasis of bladder cancer cells in vitro and in vivo [80]. P Li et al.demonstrated that Cdr1as diminished the migration and invasion capabilities of bladder cancer cells, as well as induced cell cycle arrest. Moreover, Cdr1as significantly inhibits the growth of tumor xenografts in nude mice [81].

### Glioblastoma

Glioblastoma is the most common intracranial tumor, and is one of the worst prognosis cancer worldwide. Circ-FBXW7 can encode a novel protein FBXW7-185aa, and patients whose glioblastoma tissues have higher circ-FBXW7 have a longer total survival time, when compared to patients with lower circ-FBXW7. Mice implanted with U251 and U373 cells that stably overexpress circ-FBXW7 exhibited much lower tumorigenicity and a longer lifetime [51]. Another protein-translating circRNA is circSHPRH, which is downregulated in 81% of glioblastoma samples, and encodes a functional protein SHPRH-146aa. The protein can prolong the half-life of full length SHPRH, and reduce proliferation and the malignant phenotype. Patients with higher SHPRH-146aa expression have a better prognosis, when compared to patients with lower SHPRH-146aa expression [52]. In addition, the expression of circ-SHKBP1 is elevated in glioma-exposed endothelial cells (GECs), and promotes the viability, migration and tube formation of GECs via the miR-544a/FOXP1 or miR-379/FOXP2 pathway via the AGGF1 itself or though the PI3K/AKT and ERK 1/2 pathways [45]. Circ-TTBK2 is upregulated in glioma tissues and cells, and promotes cell proliferation, migration and invasion, but inhibits the apoptosis of glioma cells. Meanwhile, circ-TTBK2 silencing results in the smaller tumor volume and longer survival period in experiments in vivo [82].

### Other cancers

Many studies have found that circRNAs also play an important role in the pathogenesis of many other tumors. For instance, the silencing of circRNA_100290 would induce G1/S arrest, suppressing cell proliferation in vitro, and markedly inhibiting the growth of tumors in vivo [46]. Furthermore, circDOCK1 inhibits cell apoptosis via the circDOCK1/miR-196a-5p/BIRC3 axis in OSCC, and has the potential to be a biomarker and therapeutic target [83]. In osteosarcoma, elevated circUBAP2 expression can promote cell proliferation in vitro and cell growth in vivo [47]. The expression level of circ-NT5C2 is significantly correlated with the apoptosis rate, and suppresses cell proliferation and invasion in vitro and tumor volume and weight in a mice model [84]. N Deng et al. found that the expression of circRNA_0009910 is augmented in osteosarcoma cells, and correlated with cell proliferation inhibition, cell cycle arrest, and apoptosis in osteosarcoma cells [85]. In addition, circNASP silence significantly inhibits the proliferation and invasion of osteosarcoma cells, as well as induces G0/G1 stage arrest [86]. Hsa_circ_0001564 knockdown evidently impedes cell viability, represses the colony formation vitality induces G0/G1 stage arrest and promotes apoptosis [87]. Moreover, J Guarnerio et al. discovered that f-circRNAs are derived from cancer-associated chromosomal translations, which can promote tumorigenesis, and enhance cell viability and resistance to therapy [54].

## Circular RNAs as a biomarker in cancer

It is known that early detection and early treatment have a very important significance to the prognosis of tumors. As mentioned above, circRNAs function primarily upstream of various regulatory networks and signaling pathways, and contribute to the implementation of early diagnosis and early treatment. CircRNAs are abundantly expressed in various tissues, and circRNA isoforms of many human transcripts are expressed at levels comparable to the canonical linear isoforms [88]. In addition, circRNAs are characterized by covalently closed loop structures and resistant to RNA exonuclease or RNase R [89], which exhibit longer half-time and more detectable than their cognate linear RNA. Furthermore, circRNAs expression are tissue- and developmental stage-specific [90]. Like widely recognized tumor biomarkers, circRNAs can also be detected in plasma and saliva [91, 92]. Therefore, circRNAs have potential as biomarkers for cancer diagnosis.

As mentioned above, various circRNAs differentially express between tumor tissues and matched normal tissues, correlated with aggressive biological behaviors. Plasma samples as a non-invasive diagnostic method, is widely used in the clinical. Here, we summarized the latest literature on the role of circRNAs, which can be detected in plasma of patients (Table 2). X Zhu et al. observed that has_circ_0013958 was significantly elevated in all LAC tissues, cells and plasma, which was correlated to TNM stage and lymphatic metastasis. In addition, the AUC of has_circ_0013958 for LAC diagnosis was 0.815, and the sensitivity and specificity was 0.755 and 0.796, respectively [57]. F-circEA can be specifically detected in the plasma and tumor tissues of EML4-ALK-positive patients. Accordingly, F-circEA can be used as a diagnostic and therapeutic marker for patients with the EML4-ALK translocation [7]. It has been reported that the expression of has_circ_0000745 is significantly downregulated in both GC tissues and plasma samples obtained from patients with GC. In GC tissues, hsa_circ_0000745 levels are significantly correlated with tumor differentiation. Meanwhile, hsa_circ_0000745 levels in plasma obtained from GC patients are significantly correlated with the TNM stage. As shown in the receiver operating characteristic (ROC) curve generated for plasma has_circ_0000745 levels and CEA levels, the AUC was 0.775, with a sensitivity and specificity of 0.800 and 0.633, respectively [93]. In plasma and tissues obtained from GC patients, has_circ_0001017 and has_circ_0061276 were evidently reduced, and these levels were significantly associated with distal metastasis. The AUC of the quadruple combination of has_circ_0001017, has_circ_0061276 in gastric dysplasia and normal controls was 0.966, with a sensitivity and specificity of 95.5% and 95.7%, respectively [94]. Similarly, has_circ_0000520 is significantly deceased in gastric cancer tissues, plasma and gastric cancer cell lines. Has_circ_0000520 level in GC tissues is negatively correlated with TNM stage, and in GC plasma is positively associated with CEA expression. The ROC curve for plasma exhibits that the AUC was 0.8967 with the sensitivity and specificity are 82.35% and 84.44%, respectively [95]. Moreover, the expression of has_circ_0000190 are suppressed in gastric cancer tissues and plasma samples, which associated with tumor diameter, lymphatic metastasis, distal metastasis, TNM stage and CA19–9 levels. When combine has_circ_0000190 in tissues and plasma, the AUC is up to 0.775, and the sensitivity and specificity are 0.712 and 0.750 [96]. In addition, has_circ_002059 is significantly decreased in gastric tissues, and its levels in plasma from postoperative gastric cancer patients is higher than those from preoperative gastric cancer patients. The expression of has_circ_002059 are evidently correlated with several clinical factors, including distal metastasis, TNM stage, gender and age. The AUC of has_circ_002059 is 0.73 [97]. Circ-LDLRAD3 is upregulated in both pancreatic cancer tissues and plasma obtained from patients with pancreatic cancer, and the AUC value, sensitivity and specificity when combined with CA19–9 was 0.87, 0.8033 and 0.9355, respectively [70]. Z Kun-Peng et al. discovered that circPVT1 was significantly elevated in the osteosarcoma tissues, serums and chemoresistant cell lines, which was correlated with advanced Enneking stage, chemoresistance and lung metastasis. The ROC curve showed that the AUC is 0.871, and consequently circPVT1 has the potential to be a diagnosis biomarker in osteosarcoma, comparable to LDH and better than ALP [98]. S Li et al. demonstrated that numerous circRNAs show differential expression in plasma derived from patients before and after cervicectomy. Besides, more than 10,000 circRNAs are detected in plasma from patients with cervical cancer [90].

**Table 2:** Circular RNAs as plasma biomarker in cancer

circRNA	Cancer type	Expression in plasma	Clinical correlation	ROC curve			References
				AUC	sensitivity	Specifity	
Hsa_circ_0013958	LAC	up	TNM stage, lymphatic metastasis	0.815	0.755	0.796	[57]
F-circEA	NSCLC	up	–	–	–	–	[7]
Hsa_circ_000745	GC	down	tumor differentiation	0.775	0.800	0.633	[93]
Hsa_circ_0001017 /hsa_circ_0061276	GC	Down	distal metastasis	0.966	0.955	0.957	[94]
Hsa_circ_0000520	GC	Down	TNM stage, CEA expression	0.8967	0.8235	0.8444	[95]
Hsa_circ_2059	GC	Down	distal metastasis, TNM stage, gender and age	0.73	–	–	[97]
Circ-LDLRAD3	Pancreatic cancer	Up	venous invasion, lymphatic invasion	0.87	0.8033	0.9355	[70]
CircPVT1	OS	Up	advanced Enneking stage, chemoresistance, lung metastasis	0.871	–	–	[98]
Circ-KLDHC10	CRC	Up	–	–	–	–	[99]
Circ-PDE8A	PDAC	Up	duodenal invasion, vascular invasion, T factor or TNM stage	–	–	–	[71]

Exosomes are small membrane vesicles secreted by various cell, as well as contain disease-specific protein and nucleic acid. Recent studies have found that circRNAs are abundant in exosomes, and may serve as a new class of exosome-based biomarker. Y Li et al. discovered that more than 1000 circRNAs were identified in the human serum exosomes [99]. Compared to healthy donors, 67 circRNAs were absent and 257 new circRNAs were discovered in CRC patients. Based on serum exosome RNA sequencing (RNA-seq) datasets, the expression of circ-KLDHC10 was upregulated in serum obtained from colorectal cancer (CRC) patients. Circ-PDE8A is abundant in exosomes of circ-PDE8A overexpressing cells, and it can regulate the expression of MACC1 and MET in vitro. The blood exosome circ-PDE8A is existed in the plasma of PDAC patients, and the expression of exosomal circ-PDE8A was associated with duodenal invasion, vascular invasion, T factor or TNM stage [71]. High quality circRNA candidates are detected in the exosomes of colon cancer cell lines. Moreover, the relative circRNA levels are not associated with their linear mRNA host genes in exosomes [100]. These studies show that serum exo-circRNA (circRNA in exosomes) has potential to be a circulating biomarker for cancer diagnosis. In addition, there is database, exoBase (http://www.exorbase.org/), which provides the expression level and possible original tissues of circRNAs in human blood exosomes, triggering the discovery and research of exo-circRNAs.

## Conclusions

In current study,it is a hot topic about the role of circRNAs in diseases. With the development of next-generation sequencing technologies and other detection technologies, increasingly circRNAs differentially expressed between disease states and normal states can be detected. As a type of disease with high morbidity and mortality, the tumor is a serious threat to human health. Recently, many studies have found that circRNAs play a crucial role in the development of multiple tumors. CircRNAs are in equilibrium under the normal state of the body, and when carcinogenic circRNAs are up-regulated or cancer-suppressing circRNAs are downregulated, tumors will form. The main mechanism of circRNAs in tumors is the miRNA sponge, which acts through the circRNA-miRNA-mRNA regulatory networks. Since Ago2 is one of the major members of RNA-induced silencing complex (RISC), the Ago2 immunoprecipitation assay is widely used to discover the miRNA sponge function of circRNA. CircRNAs regulate the downstream mRNA expression of oncoproteins, tumor suppressor proteins and cell cycle related proteins. However, most circRNAs do not function as microRNA sponges because most of these molecules contain fewer miRNA binding sites [5]. In addition, circRNAs can bind to proteins and function at the transcriptional, post-transcriptional, and translational levels. Interestingly, endogenous circRNAs have recently been found to encode protein through the 5′ cap-independent translation and play an important role in gliomas, such as circFBXW7 [51] and circSHPRH [52]. Some databases can be used for the prediction of protein-translating circRNAs, such as: circRNADb (http://202.195.183.4:8000/circrnadb/circRNADb.php), circPro (http://bis.zju.edu.cn/CircPro). Further research on the translational function of tumor-associated circRNAs is of great importance for the study of tumorigenesis mechanisms. The formation mechanism of circRNA has been studied more thoroughly, but the mechanism of its degradation remain unclear. Some studies have found that circRNAs are rich in extracellular vesicles [101] and exosomes [99], which may be one of the degradation mechanisms. Hence, further studies on the degradation mechanism of circRNAs are needed. A large number of studies have shown that circRNA is differentially expressed in the plasma of patients and normal subjects, and its tendency is the same as that between tumor tissues and adjacent normal tissues, which has important diagnostic value. However, the research and application of circRNAs in targeted therapy are few. The development direction, including antagonizing circRNA function by siRNA, anti-sense oligonucleotides and CRISPR-Cas9-mediated genome editing, promoting circRNA function by a minigene construct [102].

## Abbreviations

ADAR1: Adenosine deaminase1
AUC: Area under curve
BCa: Bladder cancer
ceRNA: Competing endogenous RNA
circRNA: Circular RNA
CRC: Colorectal cancer
GC: Gastric cancer
HCC: Hepatocellular carcinoma
IRES: Internal ribozyme entry site
LAC: Lung adenocarcinoma
NSCLC: Non-small cell lung cancer
ORF: Open reading frame
OS: Osteosarcoma
OSCC: Oral squamous cell carcinoma
PDAC: Pancreatic ductal adenocarcinoma
RBP: RNA-binding proteins;
ROC: Receiver operating characteristic
TNBC: Triple negative breast cancer
